# Evaluation of Video-Based Instruction and a 360° Virtual Reality Module on Personal Protective Equipment Competency and Infection Prevention in Healthcare Settings: A Quasi-Experimental Study

**DOI:** 10.3390/healthcare14101266

**Published:** 2026-05-07

**Authors:** Abdullah Alharbi, Ghareeb Bahari

**Affiliations:** 1 Community and Psychiatric Mental Health Nursing Department, College of Nursing, King Saud University, Riyadh 11421, Saudi Arabia; abdmalharbi@ksu.edu.sa; 2 Nursing Administration and Education Department, College of Nursing, King Saud University, Riyadh 11421, Saudi Arabia

**Keywords:** personal protective equipment, healthcare workforce training, virtual reality, infection prevention, patient safety, clinical competency

## Abstract

**Highlights:**

**What are the main findings?**

- Both VR-based and video-based instructional approaches significantly improved participants’ competency in PPE use, supporting enhanced infection prevention practices in clinical settings.
- VR-based training demonstrated slightly higher post-intervention competency scores and was associated with elevated levels of learner confidence and satisfaction, indicating its potential to strengthen healthcare workforce preparedness and promote safe patient care practices.

**What are the implications of the main findings?**

- VR-based learning offers added value as a supplementary training strategy to enhance infection prevention and procedural competency, supporting patient safety and healthcare workforce preparedness.
- Integrating VR with traditional training approaches may enhance engagement and better prepare learners for safe and effective clinical practice, supporting improved healthcare delivery.

**Abstract:**

**Background**: The use of personal protective equipment (PPE) is essential to ensure infection prevention, healthcare worker safety, and reduce healthcare-associated infections in clinical settings. Innovative training approaches, such as virtual reality (VR), may enhance competency and clinical preparedness compared with traditional video-based instruction, supporting safer patient care practices. **Methods**: Participants were 84 students, with 42 participants in each group. The study was conducted at a public university in Saudi Arabia. The video-based group viewed a standardized educational video on PPE procedures, whereas the VR group engaged in a 13-min immersive 360° training module. Both groups completed pre- and post-intervention assessments to evaluate PPE competency. Primary outcomes included the PPE Knowledge Questionnaire and the Satisfaction and Self-Confidence in Learning Scale scores. Statistical analyses were conducted using SPSS. **Results**: Both instructional approaches significantly improved PPE-related competency, supporting infection prevention in clinical settings. The VR group demonstrated slightly higher post-intervention scores than the video-based group (*p* < 0.001). The magnitude of this difference was large (Cohen’s d ≈ 0.87; partial η^2^ = 0.163), indicating a practical advantage of VR-based instruction over video-based learning. High satisfaction and self-confidence were reported among VR participants, indicating positive perceptions of clinical preparedness, although these were not significantly associated with competency gains. **Conclusions**: Both VR- and video-based training significantly enhanced PPE competency, supporting improved infection prevention practices. VR demonstrated a marginal advantage and was associated with high levels of satisfaction and confidence, highlighting its value as an effective training approach to strengthen healthcare workforce preparedness and patient safety.

## 1. Introduction

Infection prevention and control represent a critical competency domain in healthcare practice, directly influencing patient safety and quality of care. Healthcare-associated infections continue to pose a substantial global burden, contributing to prolonged hospital stays, increased morbidity, and preventable mortality [1]. The correct use of personal protective equipment (PPE), including appropriate donning and doffing of gloves, gowns, masks, and face shields, is a fundamental component of standard and transmission-based precautions essential for preventing pathogen transmission [2]. While PPE is a physical defense against pathogens, digital tools and AI are increasingly vital in managing the “infodemic” of misinformation that often accompanies outbreaks, and can impact healthcare worker compliance and infection prevention strategies [3]. Errors in PPE procedures place both patients and healthcare workers at risk, a challenge highlighted during the COVID-19 pandemic when breaches in PPE technique were reported even among trained clinical personnel worldwide [4]. Ensuring that learners acquire accurate and clinically applicable PPE competency prior to clinical exposure is therefore essential to strengthening infection prevention practices and safeguarding patient outcomes within healthcare systems.

Traditional approaches to teaching procedural skills, including face-to-face instructor demonstrations, mannequin-based simulation, and video-based instruction, have demonstrated value but also present limitations in preparing learners for real-world clinical practice. Despite its accessibility and cost-effectiveness, video-based instruction delivers procedural content in a passive, non-interactive format, which may limit engagement and reduce the transfer of skills required for safe and effective patient care [5]. From a cognitive perspective, procedural competency is enhanced when learners actively rehearse tasks within immersive and contextually relevant environments, conditions that two-dimensional formats cannot fully replicate [6]. Virtual reality (VR) refers to a computer-generated, interactive three-dimensional environment that enables users to engage with simulated scenarios in realistic settings, typically using head-mounted displays or other technological devices. In healthcare contexts, VR allows for immersive, controlled simulation of clinical settings, supporting both experiential learning and therapeutic applications [7]. VR-based training addresses these gaps by providing immersive three-dimensional environments in which learners can observe, practice, and repeat clinical procedures in a safe and standardized setting [8].

Beyond procedural training, VR has also been applied consistently across broader clinical contexts, including physical rehabilitation and mental health interventions. In rehabilitation, VR facilitates repetitive, task-oriented exercises that promote motor recovery and functional improvement [9]. In mental health settings, it enables controlled exposure and therapeutic interventions for conditions such as anxiety disorders and post-traumatic stress disorder [10]. By enhancing experiential learning and simulating real clinical workflows, VR has the potential to improve clinical preparedness, reduce procedural errors, and support infection prevention practices, contributing to improved healthcare delivery and patient safety outcomes [11].

Findings from multiple meta-analyses have consistently supported the effectiveness of VR-based simulations across multiple domains of nursing competence. Researchers synthesized 12 randomized controlled trials and reported significant VR-associated improvements in nursing knowledge scores [12]. This finding was corroborated by prior research, in which authors identified that short, self-guided sessions using low- to moderate-immersion levels were the most effective [13]. Other researchers extended these findings by demonstrating that VR significantly enhanced not only theoretical knowledge but also practical skills, skill retention, and learner satisfaction relative to traditional instruction [11]. In addition, significant improvements in overall nursing competency were reported [14], while others, in a meta-analysis of 769 nursing students [15], confirmed the capacity of VR-based simulation to improve both knowledge and clinical skills. These studies position VR as a robust instructional modality in nursing education, while acknowledging persistent heterogeneity in study designs, platforms, and outcome measures.

Procedure-specific studies have explored a range of core nursing skills, with VR-trained students consistently demonstrating superior or equivalent knowledge and skill performance across domains, including intravenous therapy, nasogastric tube feeding, urinary catheterization, and blood transfusion [16,17,18]. VR has also been applied in infection control and PPE training. A VR-based infection control simulation resulted in significantly greater improvements in performance and self-efficacy than controls [19]. Game-based VR simulations also effectively improved nursing students’ PPE knowledge and problem-solving abilities in an infection control context [20].

Most existing studies have focused primarily on patient-related outcomes or general infection control practices, with less emphasis on standardized PPE donning and doffing sequences that are critical for preventing transmission in clinical settings. Additionally, limited evidence on structured training and competency assessment highlights a gap in preparing healthcare learners for safe clinical practice. Therefore, this study aimed to evaluate the effectiveness of VR-based training in improving PPE competency compared with video-based instruction, and to assess learner satisfaction and self-confidence in relation to perceived clinical readiness. The findings are expected to inform evidence-based training strategies, enhance PPE-related competency, and support the integration of innovative technologies into healthcare training programs to improve patient safety and infection prevention outcomes.

## 2. Materials and Methods

### 2.1. Study Design and Setting

This study employed a quasi-experimental pretest–posttest design with two parallel training groups to evaluate the effects of VR-based instruction on PPE competency relevant to infection prevention in clinical practice. Participants were assigned to either a video-based or VR-based training group. Both groups completed a baseline competency assessment prior to the intervention (pretest) and the same assessment immediately following the intervention (posttest) to measure changes in clinically relevant knowledge and preparedness. The study was conducted within an undergraduate nursing program at a public university designed to prepare students for clinical practice. The curriculum consists of eight academic semesters followed by a one-year clinical internship, integrating theoretical instruction with hands-on training in fundamental nursing skills, infection prevention, and patient safety. Students are introduced to standard precautions and appropriate PPE use through classroom instruction, simulation-based skills laboratories, and supervised clinical placements, ensuring alignment with healthcare system requirements and safe patient care practices.

### 2.2. Sampling Process

The study sample consisted of undergraduate nursing students recruited using convenience sampling from selected courses within the program, representing future healthcare providers. Participants were equally allocated to either the video-based training group or the VR-based training group. Students were eligible if they were enrolled in the nursing program and had not previously received formal clinical training in PPE procedures, ensuring assessment of baseline competency relevant to infection prevention. Participation required engagement in laboratory-based learning and VR training sessions designed to simulate clinical practice. Students reporting intolerance to VR environments, such as severe motion sickness or dizziness, were excluded to ensure safety and feasibility of the training intervention.

The required sample size was estimated using G*Power 3.1 for an independent samples *t*-test to compare the two groups. Assuming a medium effect size (d = 0.50), a significance level of α = 0.05, a power of 0.80, and a one-tailed test, the analysis indicated that a minimum of 102 participants were required (51 per group). However, the final sample included 84 students (42 per group) due to the limited number of students enrolled in the course during the study period.

### 2.3. Study Procedures

#### Common Instruction

As part of regular course activities, all participants first attended a standardized theoretical lecture on PPE. The session lasted approximately 30 min, was identical for all participants, and served as background instruction prior to the study interventions. Table 1 lists the topics covered.

The VR group received training through an immersive 360° VR instructional module, while the comparison group received instruction through a standard educational video demonstrating the same PPE procedures. Additional details are provided below.

1. Video Instruction Group. Participants assigned to the video-based training group viewed a standardized educational video demonstrating correct PPE procedures aligned with infection prevention guidelines in clinical practice. The video presented the same PPE donning and doffing sequences as the VR module, ensuring consistency in training content across both modalities. It illustrated the appropriate sequence of PPE application and removal, including hand hygiene, gown application, mask placement, glove use, safe removal techniques, and proper disposal procedures essential for preventing pathogen transmission. Unlike the VR training, the video was delivered via a conventional screen without interactive or immersive elements, limiting opportunities for experiential learning. No additional instructor-led demonstration or hands-on practice was provided, reflecting a passive learning approach commonly used in healthcare training. Apart from the mode of delivery, the educational content, learning objectives, and duration were standardized across both groups to ensure comparability of training effects.
2. VR Instruction Group. The VR training content was developed in accordance with established PPE guidelines issued by the Centers for Disease Control and Prevention, ensuring alignment with evidence-based infection prevention standards in clinical practice. To maintain consistency across training modalities, the instructional content followed identical procedural steps to those used in the video-based instruction. Participants in the VR group engaged in a 13-min immersive 360° training module focused on correct PPE use. The VR module was delivered using a Meta Quest 2 headset (Meta Platforms, Inc., Menlo Park, CA, USA) and accessed via a VR content platform (Blinky PPE Training, Alpha Code, Inc., Tokyo, Japan). The module was observational, consisting of an immersive 360° video rather than a fully interactive virtual reality simulation. Presented from a first-person perspective, the VR training simulated clinical workflow and demonstrated the correct sequence of PPE application and removal, including hand hygiene, gown application, mask placement, glove use, safe removal techniques, and proper disposal procedures critical for preventing pathogen transmission. Participants could control basic playback functions, such as pausing and replaying the module; however, the instructional sequence was fixed and not modifiable. The module lacked interactive elements, embedded self-assessment, and real-time feedback. The 13-min duration was pre-determined by the platform and remained consistent across all participants. No additional instructor-led demonstration or hands-on practice was provided beyond the VR module, allowing evaluation of the standalone effectiveness of immersive training for clinical preparedness.

### 2.4. Instruments

#### 2.4.1. PPE Knowledge Questionnaire

PPE knowledge was assessed using a 15-item multiple-choice questionnaire developed specifically for this study. The questionnaire items were developed from established guidelines for PPE use issued by the Centers for Disease Control and Prevention and reflected the procedural steps for the correct donning and doffing of PPE. The instrument was designed to assess participants’ knowledge of key aspects of PPE use, including the correct sequence of donning and doffing, appropriate hand hygiene practices, proper mask handling, and contamination prevention principles. Content validity was assessed qualitatively through expert review. Two infection control specialists evaluated the clarity, readability, and comprehensibility of the items, and minor revisions were made based on their feedback to improve wording and ensure ease of understanding. The scale’s items had one correct response per each, resulting in a total possible score ranging from 0 to 15, with higher scores indicating greater PPE knowledge. Internal consistency was assessed using the Kuder–Richardson Formula 20 (KR-20), an appropriate reliability coefficient for dichotomously scored items. KR-20 values of 0.861 and 0.822 were obtained for the VR and video groups, respectively. Item-level psychometric properties, such as difficulty and discrimination indices, were not evaluated for this specific instrument, which may limit the depth of evidence supporting its measurement properties.

#### 2.4.2. Satisfaction and Self-Confidence in Learning

Satisfaction and self-confidence related to the VR-based learning experience were measured using the Student Satisfaction and Self-Confidence in Learning instrument developed by the National League for Nursing [https://shorturl.at/sFOnU (accessed on 30 August 2025)]. The instrument includes 13 items distributed across two subscales: satisfaction with the learning experience (five items) and self-confidence in learning (eight items). Responses were rated on a 5-point Likert scale ranging from 1 (strongly disagree) to 5 (strongly agree), with higher scores indicating greater satisfaction and confidence. The instrument has demonstrated excellent internal consistency, with reported Cronbach’s alpha values of 0.94 for satisfaction and 0.87 for self-confidence. In this study, the instrument was administered exclusively to the VR group because its items are specific to simulation-based learning contexts.

### 2.5. Data Collection Procedures

Data were collected during scheduled laboratory-based training sessions designed to simulate clinical learning environments. Prior to the intervention, participants in both groups completed a PPE competency questionnaire to establish baseline knowledge relevant to infection prevention (pretest). Following the instructional sessions, the same instrument was administered to assess post-intervention competency (posttest). In addition to the competency assessment, participants in the VR group completed the Satisfaction and Self-Confidence in Learning Scale to evaluate perceived readiness for clinical application and training effectiveness. All responses were collected anonymously using coded identifiers to ensure confidentiality and compliance with research and ethical standards.

### 2.6. Data Analysis

Data were analyzed using SPSS (v. 30; IBM Corp., Armonk, NY, USA). Before conducting the analyses, the data were examined for missing values, outliers, and normality. No missing data were identified, and potential outliers were assessed by reviewing data distributions and identifying extreme values. Normality was evaluated using the Shapiro–Wilk test, which indicated no significant deviations (*p* > 0.05). Descriptive statistics were used to summarize demographic characteristics and pretest and posttest scores. An independent-samples *t*-test was conducted to evaluate differences in post-intervention outcomes (continuous variable) between groups defined by a binary demographic characteristic (e.g., gender). Pearson’s correlation coefficient was used to assess associations among continuous variables, including age, satisfaction, self-confidence, and both video- and VR-based instruction measures. Paired *t*-tests were used to examine differences between pretest and posttest scores within each group. Additionally, analysis of covariance (ANCOVA) was conducted to compare posttest PPE knowledge scores between the video-based and VR-based instructions groups, while controlling for gender and pretest scores. Significance value was set at *p* < 0.05.

## 3. Results

### 3.1. Participant Characteristics

This study included 84 male and female nursing students aged between 20 and 22 years. Some participants were enrolled in the Fundamentals of Nursing course, whereas most were enrolled in the Infection Control course. In the video-based instruction group, the mean pretest score was 5.79 (SD = 1.76; range, 3–10). Following the intervention, the mean posttest score increased to 12.67 (SD = 1.03; range, 11–15), indicating an improvement in students’ knowledge after video-based instruction. Similarly, in the VR instruction group, the mean pretest score was 5.83 (SD = 1.99; range, 3–10). After the VR intervention, the mean posttest score increased to 13.55 (SD = 0.99; range, 11–15), suggesting an improvement in students’ knowledge following the VR-based learning experience. More details, including satisfaction and confidence levels, are presented in Table 2.

### 3.2. Baseline and Post-Intervention Characteristics Between Groups

Baseline analyses showed no significant association between age and the study groups (video-based: *p* = 0.761; VR-based: *p* = 0.33), indicating that the groups were comparable prior to the intervention. Gender showed a significant difference in the video-based group (*p* = 0.03) but not in the VR group (*p* = 0.30) (Table 3).

After the intervention, age remained not significantly associated with the groups (video-based: *p* = 0.107; VR-based: *p* = 0.67). However, sex differences remained significant only in the video-based group (*p* = 0.04) and not in the VR group (*p* = 0.25). Satisfaction (*p* = 0.22) and self-confidence (*p* = 0.16), assessed only among students in the VR-based instruction group, were not significantly associated with knowledge outcomes. Table 4 presents further details.

### 3.3. Comparison Within and Between Groups

As shown in Table 5, paired *t*-test analyses revealed a statistically significant improvement in knowledge scores in both instructional groups following the intervention. In the video-based instruction group, the mean knowledge score increased from 5.79 (SD = 1.76) at pretest to 12.67 (SD = 1.03) at posttest, representing a significant difference (t(41) = −24.10, *p* < 0.001). Similarly, in the VR-based instruction group, the mean score improved from 5.83 (SD = 1.99) at pretest to 13.55 (SD = 0.99) at posttest, which was also statistically significant (t(41) = −22.17, *p* < 0.001). These findings indicate that both instructional methods significantly enhanced students’ knowledge of PPE procedures, with slightly higher post-intervention scores observed in the VR-based instruction group than in the video-based instruction group.

In Table 6, gender was included as a covariate in the ANCOVA model alongside pre-test scores. The results indicated that gender did not significantly predict post-test PPE knowledge scores (F(1,80) = 1.412, *p* = 0.238, partial η^2^ = 0.017), suggesting that the training effect was not confounded by participant sex. Importantly, the group effect remained statistically significant after controlling for both pre-test scores and sex (F(1,80) = 15.879, *p* < 0.001, partial η^2^ = 0.166), with the VR group demonstrating superior post-test performance compared to the video group (M = 13.55, SD = 0.99 vs. M = 12.67, SD = 1.03). The effect size (Cohen’s d = 0.87) further indicates a large practical difference between groups.

### 3.4. Satisfaction and Self-Confidence in the VR-Based Learning Experience

Table 7 presents descriptive and correlation statistics for satisfaction and self-confidence among participants in the VR-based training group, reflecting perceptions of training effectiveness and clinical preparedness. The analysis was limited to post-intervention data to evaluate the perceived impact of immersive training on readiness for clinical application. Participants reported high levels of satisfaction (M = 22.57, SD = 1.25; range = 19–25) and self-confidence (M = 35.54, SD = 2.06; range = 31–40), indicating positive acceptance of VR as a training approach for infection prevention practices. However, no significant association was observed between satisfaction, self-confidence, and post-intervention competency scores (*p* > 0.05), suggesting that perceived confidence may not directly correspond to measurable competency gains.

## 4. Discussion

This study examined the effectiveness of VR-based instruction compared with video-based instruction in improving PPE knowledge. Overall, the findings showed that both instructional approaches significantly improved participants’ knowledge, with slightly higher posttest scores observed in the VR group. These results suggest that technology-enhanced learning methods play an important role in supporting nursing education, particularly in infection prevention and clinical procedures [21]. The findings that VR-based instruction matches or slightly exceeds traditional methods align with broader scholarly debates on whether digital interventions produce comparable or superior effects to conventional face-to-face approaches [22]. Previous research has also reported that digital learning approaches can enhance learner engagement and knowledge acquisition in healthcare education [18]. Further, technology can support experiential learning by allowing learners to interact with realistic clinical environments [23].

The results in Table 2 indicate that both instructional methods improved participants’ knowledge of PPE procedures. Participants in the video-based instruction group demonstrated a clear increase in posttest scores compared with pretest scores. This finding confirms that video-based learning remains an effective strategy for delivering clinical education and demonstrating procedural skills [24]. Educational videos allow repeatedly observing clinical practice, which can reinforce theoretical knowledge and improve understanding of step-by-step procedures [25,26]. However, participants in the VR group achieved slightly higher posttest scores than those in the video group. This may be attributed to the immersive 360° format, which provides good perspective and a greater sense of spatial presence compared to traditional video. Such immersive viewing may support clearer visualization of procedures and the sequence of clinical actions, potentially enhancing understanding and knowledge retention [27,28].

Baseline analyses showed that age was not significantly associated with the study groups, indicating that participants were comparable before the intervention. This similarity between groups strengthens the reliability of the study results and suggests that the observed improvements in knowledge were likely due to the instructional methods rather than demographic differences. Although sex differences were identified in the video-based group, they were not significantly associated with learning outcomes in the VR group. Previous research has also indicated that demographic factors such as age and sex often have limited influence on learning outcomes in simulation-based education [28]. Satisfaction and confidence in the VR group were also not significantly associated with posttest scores. This suggests that, although students may feel satisfied and confident following VR-based learning, these subjective perceptions do not always reflect objective learning outcomes [29].

Paired *t*-test results demonstrated significant improvements in knowledge scores in both groups after the intervention. These findings indicate that both video- and VR-based instruction are effective methods for teaching PPE procedures. Video-based instruction has long been used in nursing education as a practical and accessible approach for demonstrating clinical skills and reinforcing theoretical concepts [24]. However, the greater improvement observed in the ANCOVA test for the VR group suggests that immersive technologies provide additional learning benefits [8]. In this study, these benefits are likely related to the VR format, which offers an enhanced spatial presence, increase attentional engagement, and support more intuitive understanding of procedural flow.

Participants in the VR-based instruction reported high levels of satisfaction and self-confidence following the learning experience. Immersive learning technologies positively influence participants’ perceptions of their learning environment and their readiness to perform clinical procedures [30,31]. VR-based instruction often creates engaging and interactive learning experiences that increase motivation and interest in the learning process [32]. The ability to visualize procedures in a realistic environment may also increase confidence in their understanding of clinical tasks [18]. Despite these positive perceptions, no significant relationship was found between satisfaction, confidence, and knowledge scores. This suggests that, while students may enjoy the VR learning experience and feel more confident, these perceptions do not necessarily translate into higher measurable knowledge outcomes. Similar findings have been reported in other simulation-based education studies, in which satisfaction and confidence were important educational outcomes but were not always strongly correlated with academic performance [33].

In comparison with the global literature, our findings align with studies showing that VR can enhance learning outcomes and engagement in nursing education [34]. However, unlike studies reporting substantial gains, this study found only a modest advantage over video-based instruction. This variation may be related to differences in the level of immersion, instructional design, or learners’ prior exposure to digital technologies. Future research should examine the impact of varying levels of immersion, VR hardware configurations, and degrees of interactivity on learning outcomes, as these factors may influence training effectiveness and help establish standardized technical benchmarks for VR-based healthcare education. Some literature also questions whether the added cost and complexity of VR are justified when simpler methods provide comparable knowledge. In this context, formal economic evaluations are needed to assess whether the marginal gains in competency associated with VR justify its additional financial and technical demands. Overall, the present findings support the complementary use of VR alongside traditional teaching methods.

### 4.1. Study Limitations

This study has several limitations. First, the quasi-experimental design limits causal inference. Future studies should employ randomized clinical trials to minimize selection bias and strengthen the link between VR training and competency gains. Second, utilizing a convenience sample from a single institution limits generalizability. Multicenter studies involving diverse student populations and practicing healthcare professionals would increase the external validity of the findings. Third, the study only assessed immediate post-intervention outcomes. Including follow-up assessments (e.g., at 3 or 6 months) would provide critical evidence on the retention of VR-based PPE skills over time. Fourth, the current study did not achieve the sample size determined by a priori power analysis and is therefore potentially underpowered to detect between-group differences. However, the ANCOVA results demonstrated a statistically significant difference favoring the VR-based instruction, with a large effect size. These findings should therefore be interpreted as valid within the context of the present study but with caution regarding their generalizability and precision. While the observed effect suggests that VR-based instruction may offer an advantage, further research with larger, more diverse samples and broader outcome measures is needed to confirm and extend these findings rather than to establish their validity.

Additionally, satisfaction and self-confidence were assessed only in the VR group due to the simulation-specific nature of the instrument, which prevented direct comparison of learner experiences across groups. Furthermore, the exploratory analyses of associations between satisfaction, self-confidence, and competency gains may be underpowered; therefore, the absence of statistically significant relationships should be interpreted with caution. Another important limitation is the restriction of outcome assessment to a knowledge-based questionnaire, without direct observation of participants’ practical performance. Although the findings suggest improved understanding of donning and doffing procedures, the extent to which this knowledge translates into correct clinical practice remains unclear. Future studies should incorporate observational and simulation-based assessments in clinical or controlled settings to evaluate skill transfer from virtual training to real-world practice and to provide a more comprehensive measure of competency.

### 4.2. Study Implications and Future Directions

The findings of this study highlight the potential value of integrating innovative training strategies, such as VR, into healthcare education to strengthen PPE competency and infection prevention practices. Both VR- and video-based approaches significantly improved competency, indicating that digital training tools can effectively support infection control in clinical settings. However, the slightly greater gains observed with VR, along with high levels of satisfaction and confidence, suggest that immersive training may enhance engagement and experiential learning relevant to clinical performance. Therefore, integrating VR simulations alongside conventional training approaches may improve procedural understanding, support workforce preparedness, and contribute to safer patient care in real-world healthcare environments. From a practical perspective, future training programs should consider adopting blended, multimodal approaches integrating video-based instruction, VR simulation, and hands-on practice. Such approaches may support the complementary strengths of each modality enhance competency development, skill retention, and overall healthcare workforce preparedness.

From a healthcare management and implementation perspective, improving competency and confidence in PPE use is critical for ensuring effective infection prevention and maintaining patient and healthcare worker safety. VR-based training may help bridge the gap between theoretical instruction and clinical practice by enabling learners to engage in realistic, risk-free scenarios that simulate healthcare environments. Future research should examine the long-term impact of VR-based training on skill performance, knowledge retention, and clinical behavior, as well as its effectiveness compared with other simulation-based approaches. Future studies should examine the integration of VR-based training into routine healthcare education and institutional training programs and evaluate technological barriers and implementation challenges across diverse clinical settings. Additionally, healthcare educators and administrators should consider feasibility, cost-effectiveness, and system readiness when integrating VR technologies to ensure sustainable implementation and optimal impact on healthcare delivery and patient safety.

## 5. Conclusions

The findings of this study indicate that digital training tools can effectively support healthcare education and clinical practice, with VR-based training demonstrating greater improvements in PPE competency compared with video-based instruction. VR enables learners to observe and rehearse procedural steps within a simulated clinical context, facilitating competency development in infection prevention and adherence to safety protocols. Although VR-based training demonstrated only a marginal advantage over video-based instruction, its higher cost and technical requirements should be considered, and future studies incorporating formal cost-effectiveness analyses are needed to guide implementation decisions. Furthermore, VR represents a valuable supplementary training strategy for infection prevention and control, particularly in settings with limited access to clinical placements or hands-on practice. Further interventional research is recommended to evaluate the impact of VR on clinical performance, knowledge retention, and healthcare workforce preparedness, as well as its role in improving patient safety outcomes.

## Figures and Tables

**Table 1:** Topics covered in the PPE instruction.

Topic	Content Description
Types of PPE	Overview of common PPE (gloves, gowns, masks/respirator, etc.) and their appropriate use in healthcare settings
Donning Procedures	Correct sequence and technique for putting on PPE, including hand hygiene and proper fitting of equipment
Doffing Procedures	Safe sequence for removing PPE to minimize self-contamination and proper disposal of PPE
Infection Prevention Principles	Basic concepts of infection transmission, standard precautions, and the role of PPE in preventing disease spread

**Table 2:** Instruction methods scores by groups.

Variable	Group (n)	Pretest Scores	Posttest Scores
Instruction Methods	Video-based (42)	M = 5.79 SD = 1.76	M = 12.67 SD = 1.03
	VR-based (42)	M = 5.83 SD = 1.99	M = 13.55 SD = 0.99
Satisfaction		-	M = 22.57 SD = 1.25
Confidence		-	M = 35.54 SD = 2.06

**Table 3:** Pretest scores between the groups.

Variable	Video-Based Instruction (n = 42)	*p*-Value	VR-Based Instruction (n = 42)	*p*-Value
Age	−0.048	0.761	0.153	0.33
Gender (%)		0.03 *		0.30
Male	69		69	
Female	31		31	

* *p* < 0.05; Pearson coefficient assessed age–pretest scores associations; independent *t*-tests examined gender differences.

**Table 4:** Posttest scores between the groups.

Variable	Video-Based Instruction (n = 42)	*p*-Value	VR-Based Instruction (n = 42)	*p*-Value
Age	−0.253	0.107	−0.067	0.67
Gender (%)		0.04 *		0.25
Male	69		69	
Female	31		31	
** Satisfaction	-	-	0.193	0.22
** Confidence	-	-	−0.222	0.16

* *p* < 0.05; ** Pearson correlations were used to assess associations between age, satisfaction, confidence, and posttest scores; independent samples *t*-tests were used to examine differences by gender.

**Table 5:** Comparison of pre- and post-test scores within each group (Paired *t*-test).

Group	Pre-Test Mean (SD)	Post-Test Mean (SD)	Mean Difference	df	t-Value	*p*-Value
Video-based instruction	5.79 (1.76)	12.67 (1.03)	−6.88	41	−24.09	<0.001 *
VR-based instruction	5.83 (1.99)	13.55 (0.99)	−7.71	41	−22.16	

* Significant level at <0.05; Paired *t*-test was used to compare pre-test and post-test knowledge scores within each group.

**Table 6:** ANCOVA for posttest scores by instructional group (Controlling for pretest).

Source	Sum of Squares	df	Mean Square	F	*p*-Value	Partial η^2^
Gender	1.442	1	1.442	1.412	0.238	0.017
Pretest Score	0.494	1	0.494	0.483	0.489	0.006
Group	16.222	1	16.222	15.879	<0.001 *	0.166
Error	81.730	80	1.022			
Total (Corrected)	100.036	83				

* Significant level at <0.05; Model R^2^ = 0.183 (Adjusted R^2^ = 0.152).

**Table 7:** Nursing students’ satisfaction and self-confidence following the VR-based instruction (Post-test) (n = 42).

Variable	Mean (SD)	Min–Max	r (with Post-Test Score)	*p*-Value
Satisfaction	22.57 (1.25)	19–25	0.193	0.220
Confidence	35.54 (2.06)	31–40	−0.222	0.159

## Data Availability

The datasets generated and analyzed during the current study are not publicly available due to privacy and ethical restrictions, but are available from the corresponding author upon reasonable request.

## Abbreviations

PPE	personal protective equipment
VR	Virtual reality
